# Modal beam splitter: determination of the transversal components of an electromagnetic light field

**DOI:** 10.1038/s41598-017-08657-9

**Published:** 2017-08-22

**Authors:** Michael Mazilu, Tom Vettenburg, Martin Ploschner, Ewan M. Wright, Kishan Dholakia

**Affiliations:** 1 SUPA, School of Physics and Astronomy, University of St Andrews, St Andrews, Fife KY16 9SS UK; 2 College of Optical Sciences, The University of Arizona, 1630 East University Boulevard, Tucson, AZ 85721-0094 USA

## Abstract

The transversal profile of beams can always be defined as a superposition of orthogonal fields, such as optical eigenmodes. Here, we describe a generic method to separate the individual components in a laser beam and map each mode onto its designated detector with low crosstalk. We demonstrate this with the decomposition into Laguerre-Gaussian beams and introduce a distribution over the integer numbers corresponding to the discrete orbital and radial momentum components of the light field. The method is based on determining an eigenmask filter transforming the incident optical eigenmodes to position eigenmodes enabling the detection of the state of the light field using single detectors while minimizing cross talk with respect to the set of filter masks considered.

## Introduction

What defines an optical beam and what defines a superposition of beams is a matter of convention. From the perspective of Maxwell’s equations, there is no difference due to the linearity of the equations. Nevertheless, a small number of beam families is predominantly used in optics because of their ease of description and their inherent symmetries. In practice, a non-conventional orthogonal basis is often better suited to specific situations. Although any beam can be mathematically decomposed in any basis, to our knowledge, an experimental equivalent is not yet demonstrated. Theoretical approaches have been proposed ranging from cascaded diffractive optics systems^1, 2^, to arrays of feedback based beamsplitters^3^ and to the use of the cosine-sine decomposition^4^. The efficient and unambiguous determination of the exact transversal components (eigenstates) of a light field has come to the fore as a major topic in recent years. This has particularly been the case with light fields possessing orbital angular momentum for applications in information processing. The optical solution we propose and study here acts as a multi-output beam splitter where each output corresponds to one of the basis set beams. Each basis set is associated with a different beam splitter, essentially acting as a mode converter^5^. The proposed configuration aims for a zero false positive rate so that, neglecting experimental limitations and detection efficiency, photons detected at one output are irrevocable evidence of the presence of the mode associated with that output port. We demonstrate its use for determining the transversal modes of input Laguerre-Gaussian modes but stress the approach is generic and can be extended to other orthonormal basis sets.

For such a decomposition to be unambiguous, we need the individual beams in the basis set to be orthogonal to one other. Indeed, just like a superposition of linearly polarized and circularly polarized light, beams that are not orthogonal to one other share a co-linear part that cannot be separated unambiguously. Examples of such orthogonal beam families are Laguerre-Gaussians^6^, eigenfunction representations of the Debye-Wolf diffraction integral^7^, and more generally optical eigenmode beams^8^. The first two examples correspond to specific optical setups while the optical eigenmode approach can be used in a more general optical setup^9^.

Given a decomposition basis set, we search for a complex-values filter or mask that can split and route the different components of any incident beam in different spatial directions. Such a beam splitter will in effect realize a transverse-mode tomography, capable of measuring the individual modal components of a beam. This question can be answered using different approaches ranging from a generic one-dimensional general beam splitter^3^, to more specific versions aimed at distinguishing the orbital angular momentum in beams^10–12^. Another possible approach for orbital angular momentum beams are the log-polar transformation^13, 14^ which transform the azimuthal phase variation to a linear variation. In these cases it is also possible to simply look at the far field diffraction patterned from either engineered transmission masks^15, 16^ or random diffusers^17^ to detect and distinguish between different orbital angular momentum values. These approaches are not necessarily suitable for the single photon case where the measure of the orbital angular momentum needs to be achieved using a single measure and a detector^18–21^. A more generic approach, suitable for other modes than orbital angular momentum modes, is presented by the optical implementation of the random diffuser method^22^ or by the superposition of carrier frequency modulated single beam masks^23^. The first approach is based on a black-box genetic algorithm approach while the second approach is restricted to spectrally non-overlapping modes ultimately limiting its cross talk performance, as illustrated in the results section. Here, we present a generic two-dimensional transverse mode converter that is able to decompose a beam into any arbitrary basis, with high accuracy and low cross talk.

The paper is structured as follows: In the first part, we introduce the notion of optical eigenmasks and describe their use to achieve optical transverse mode/state tomography. Numerically, we use the cases of Hermite-Gaussian and Laguerre-Gaussian beams to illustrate the detection of the two-dimensional transversal state of the light field. We discuss the important implications of this approach for single photons. This method is implemented experimentally to measure the azimuthal and radial indices of pure Laguerre-Gaussian beams.

## Results

The main result of this paper is the introduction of the eigenmask which expands the concept of optical eigenmodes^9^ and enable the modal decomposition of an input beam with respect to a chosen transverse basis set, e.g. Hermite-Gaussian or Laguerre-Gaussian modes. Each individual mode is directed by the spatial filter mask towards a finite sized detector in the output plane. More precisely, we discuss how the transverse mode content of an input beam can be efficiently measured by an array of point detectors located on the output plane and positioned judiciously.

In the following, we consider monochromatic solutions of Maxwell’s equations in free-space. More precisely, we start by formally defining the relationship between the electromagnetic fields in an output region of interest (ROI) and the input fields. Indeed, the output field can be written with the help of a linear functional ${{\mathcal{P}}}_{A}$ relating *E* the electric scalar field defined on an input surface *A* for linearly polarized light

1 $$F={{\mathcal{P}}}_{A}(E)$$

where *F* is the electric field in the output planes. In free-space, this relationship corresponds to a diffraction integral. In general, this relationship will include the effects of the different optical elements in the path between the input and output plane. In an experimental setting, this linear relationship can be measured using spatial light modulators determining the transmission matrix of the optical system^24, 25^. Crucially, this relationship is linear and maintains this property after multiplication of the input fields *E*(**r**) with a spatial transmission filter mask *M*(**r**). The effect of a probe mask *M*_*i*_ can be represented as ${F}_{i}={{\mathcal{P}}}_{A}({M}_{i}E)$. More generally, a linear superposition ∑_*i*_ *a*_*i*_ *M*_*i*_ of *N*_*f*_ masks yields an output field

2 $$F=\sum _{i}{a}_{i}{{\mathcal{P}}}_{A}({M}_{i}E)=\sum _{i}{a}_{i}{F}_{i}$$

with *i* = 1, 2, … *N*_*f*_. The total intensity of the output field *F* integrated over a region of interest (ROI) defined on the output plane is given by

3 $${m}^{(I)}(F)={\int }_{ROI}d\sigma \,F{F}^{\ast }.$$

This integral can be written as a general quadratic matrix having the form

4 $${m}^{(I)}(F)=\sum _{jk}{a}_{j}^{\ast }{R}_{jk}{a}_{k}$$

where the elements *R*_*jk*_ of the (*N*_*f*_ × *N*_*f*_) Hermitian matrix are defined by

5 $${R}_{jk}={\int }_{ROI}d\sigma \,{F}_{k}{F}_{j}^{\ast }.$$

In a manner analogous to the optical eigenmode method we define an “eigenmask” as a linear superposition of the probe masks

6 $${{\mathbb{M}}}^{\mu }=\frac{1}{\sqrt{{\lambda }^{\mu }}}\sum _{j}({v}_{j}^{\mu }){M}_{j}$$

where ${\sum }_{k}{R}_{jk}{v}_{k}^{\mu }={\lambda }^{\mu }{v}_{j}^{\mu }$, *λ* ^*μ*^ is an eigenvalue labeled by the index *μ*, and ${v}_{j}^{\mu }$ the associated eigenvector. More specifically, the eigenvalues of *R*_*jk*_ are real and can be ordered according to their magnitude *λ*_*μ*_ ≤ 1, while its eigenvectors ${v}_{j}^{\mu }$ are orthonormal and complete with respect to the set of masks used in the superposition (this means that the set of eigenvectors will take into account all degrees of freedom accessed by the probing set of masks). The output vector field ${{\mathbb{F}}}^{\mu }={{\mathcal{P}}}_{A}({{\mathbb{M}}}^{\mu }E)$ produced by the *μ* ^*th*^ eigenmask corresponds to an optical eigenmode, and has a intensity reduced by the factor *λ*_*μ*_ with respect to the intensity of the input field integrated over the surface *A*. The eigenvalues therefore relate to the transmission coefficient of the input field after being acted on by the eigenmask, propagated across free-space, and then restricted to the ROI. The principal eigenmode, corresponding to the largest eigenvalue, therefore maximizes the intensity delivered to the ROI (and optimizes detection efficiency). Furthermore, it is possible to decompose an unknown target field *T* defined on the output plane using its projection onto the optical eigenmodes ${c}^{\mu }={\int }_{ROI}d\sigma T\cdot {{\mathbb{F}}}^{\mu \ast }$. We can reconstruct the unknown field *T* from the projection using $T={{\mathcal{P}}}_{A}({c}^{\mu }{{\mathbb{M}}}^{\mu }F)$, that is, we can determine the mask that best transforms the input field *F* into the target output field *T*. This is equivalent to the methods used for the generation of arbitrary complex wave fronts. We remark that perfect target field reconstruction requires that the optical degrees of freedom accessible by the masks include, in a distributed way, the transformation between input and output field. This is not necessarily the case. If, for example, the optical system including all masks considered do not change the polarization it is not possible to reconstruct a target with an orthogonal polarization to the input field.

So far, we have confined our attention to a single input field *E*. The second step of our method is to consider a family of *N* input fields or modes ${E}_{\ell }$, $\ell =1,2,\ldots N$, such as Laguerre-Gaussian, Hermite-Gaussian, or Bessel modes, where the index $\ell$ corresponds to a placeholder for the discrete or continuous indices describing each mode of the family (Table 1 for an example). In this case, we need to define the output field ${F}_{\ell }$ as a function of the input field ${E}_{\ell }$ as

7 $${F}_{\ell }=\sum _{i}{a}_{i}{\mathcal{P}}_{A}({M}_{i}{E}_{\ell })=\sum _{i}{a}_{i}{F}_{i\ell },$$

where we have allowed for a superposition of masks *M*_*i*_. Further, we define the total intensity collected at the output over the ROI as the sum of all the intensities

8 $$\sum _{\ell }{m}^{(I)}({F}_{\ell })=\sum _{jk}{a}_{j}^{\ast }{S}_{jk}{a}_{k}$$

with

9 $${S}_{jk}=\sum _{\ell }{\int }_{ROI}d\sigma {F}_{j\ell }^{\ast }{F}_{k\ell }$$

**Table 1:** Mapping between single index $\ell$ and dual index characterizing Laguerre-Gaussian and Hermite-Gaussian modes.

Mode\ℓ	1	2	3	4	5	6	7	8	9
Laguerre-Gaussian $L{G}_{L}^{P}$	${{\rm{LG}}}_{0}^{0}$	${{\rm{LG}}}_{1}^{0}$	${{\rm{LG}}}_{2}^{0}$	${{\rm{LG}}}_{0}^{1}$	${{\rm{LG}}}_{1}^{1}$	${{\rm{LG}}}_{2}^{1}$	${{\rm{LG}}}_{0}^{2}$	${{\rm{LG}}}_{1}^{2}$	${{\rm{LG}}}_{2}^{2}$
Hermite-Gaussian $TE{p}_{x}{p}_{y}$	TE_00_	TE_01_	TE_02_	TE_10_	TE_11_	TE_12_	TE_20_	TE_21_	TE_22_

This expression is formally equivalent to the single input field case with *S*_*jk*_ replacing *R*_*jk*_. By finding the eigenvalues and eigenvectors of *S*_*jk*_ it is therefore possible to introduce eigenmasks ${{\mathbb{M}}}^{\mu }$ that deliver the maximal intensity to the ROI when illuminated by the whole family of input modes ${E}_{\ell }$. We note, however, that the parts of the optical eigenmodes associated to the different input modes ${E}_{\ell }$

10 $${{\mathbb{F}}}_{\ell }^{\mu }={\bf{P}}({{\mathbb{M}}}^{\mu }{E}_{\ell })$$

are different for each input mode labeled by $\ell$. Similarly to the case of the single beam illumination, the eigenmask ${{\mathbb{M}}}^{\mu }$ with the largest eigenvalue corresponds to mask the delivers the highest density of optical degrees of freedom to the ROI implying improved optical resolution and highest informational content^26^.

For the final step in our method, it is necessary to recognize that for any input mode ${E}_{\ell }$ it is possible to decompose a corresponding target field ${T}_{\ell }$ on the output plane into the optical eigenmodes ${{\mathbb{F}}}_{\ell }^{\mu }$. Indeed, considering a different target field for each mode $\ell$ determines the coefficients *c* ^*μ*^ describing a single mask ${\sum }_{\mu }{c}^{\mu }{{\mathbb{M}}}^{\mu }$ that best overlaps the target field ${T}_{\ell }$ when illuminated by the mode ${E}_{\ell }$. More precisely, we introduce a ROI composed by *N* Dirac delta-functions $\delta ({\bf{r}}-{{\bf{r}}}_{\ell })$ each centered at a distinct position ${{\bf{r}}}_{\ell }$ in the output plane and associated with a different mode index $\ell$. Decomposing the target field ${T}_{\ell }=\delta ({\bf{r}}-{{\bf{r}}}_{\ell })$ into the corresponding optical eigenmodes ${{\mathbb{F}}}_{\ell }^{\mu }$ determines the mask that excites only the selected Dirac delta-function $\delta ({\bf{r}}-{{\bf{r}}}_{\ell })$. The final modal splitting eigenmask ${\mathbb{W}}$ is defined by

11 $${\mathbb{W}}=\sum _{\mu }{c}^{\mu }{{\mathbb{M}}}^{\mu }$$

with

12 $${c}^{\mu }={\int }_{ROI}d\sigma \sum _{\ell }{T}_{\ell }{{\mathbb{F}}}_{\ell }^{\mu }.$$

It is the summation in the last step that defines a single mask acting across all input beams considered. Positioning a point detector at the location of the Dirac delta-function will deliver a signal only when the associated input mode of index $\ell$ is present at the input. In this way it is possible to configure a mask that redirects each of the *N* transverse mode components in an input beam towards a distinct point detector at the output plane. Due to the linearity of the process, the relative phase and amplitude relationship of the separated components is maintained. The method is summarized by algorithm 1. In the following sections we shall demonstrate numerically and experimentally that this method can be used to decompose an input field into a variety of different transverse mode components.

**Algorithm 1:**
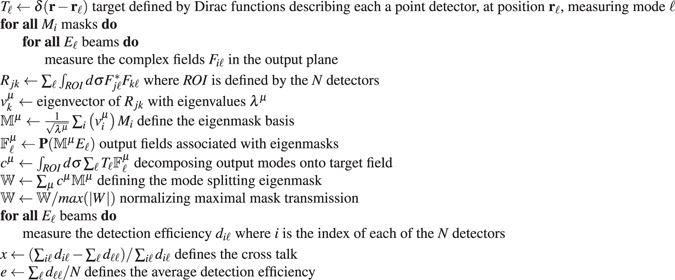
Determining the mode splitting eigenmask.

Note the parallels between our method and the concepts underlying quantum information processing. For a given input transverse mode of index $\ell$ we can configure an eigenmask ${{\mathbb{M}}}_{\ell }$ to target a given point detector on the output plane using the corresponding orthonormal optical eigenmodes ${{\mathbb{F}}}_{\ell }^{\mu }$. However, in order to measure the transverse mode content of a general input beam, it is necessary to apply all *N* eigenmasks, one for each $\ell$, simultaneously, so that we are processing the input beam in *N* ways in parallel. This is analogous to the concept in quantum information processing that many parallel realizations are being processed simultaneously. Building further upon the analogy, another key idea in quantum information is that of interference between the alternative outcomes, which in the present case manifests itself as cross talk between the point detectors at the output. This detector cross talk shall be addressed in the next section.

### Numerical simulation

To validate our approach numerically, we simulated the propagation of Laguerre-Gaussian beams ${{\rm{LG}}}_{L}^{P}$ with the azimuthal *L* and radial *P* order varying respectively from −1 to 1 and from 0 to 2. Additionally, we consider Hermite-Gaussian beams ${{\rm{TE}}}_{{p}_{x}{p}_{y}}$ with the horizontal *p*_*x*_ and vertical order *p*_*y*_ varying from 0 to 2. The propagation is simulated through a Fourier transform of the incident field corresponding to a long focal length lens in 2-f configuration. The mask is defined as a phase and amplitude mask acting on the incident field before Fourier propagation. The incident fields are discretized on a square grid. The masks taken into account correspond to all possible linear deflection masks defined for the discretization considered, this constituting a complete orthonormal basis set for the discrete system. This is possible in this case as the optical system has a finite number of modes due to finite input/output and unitary propagation.

**Figure 1:**
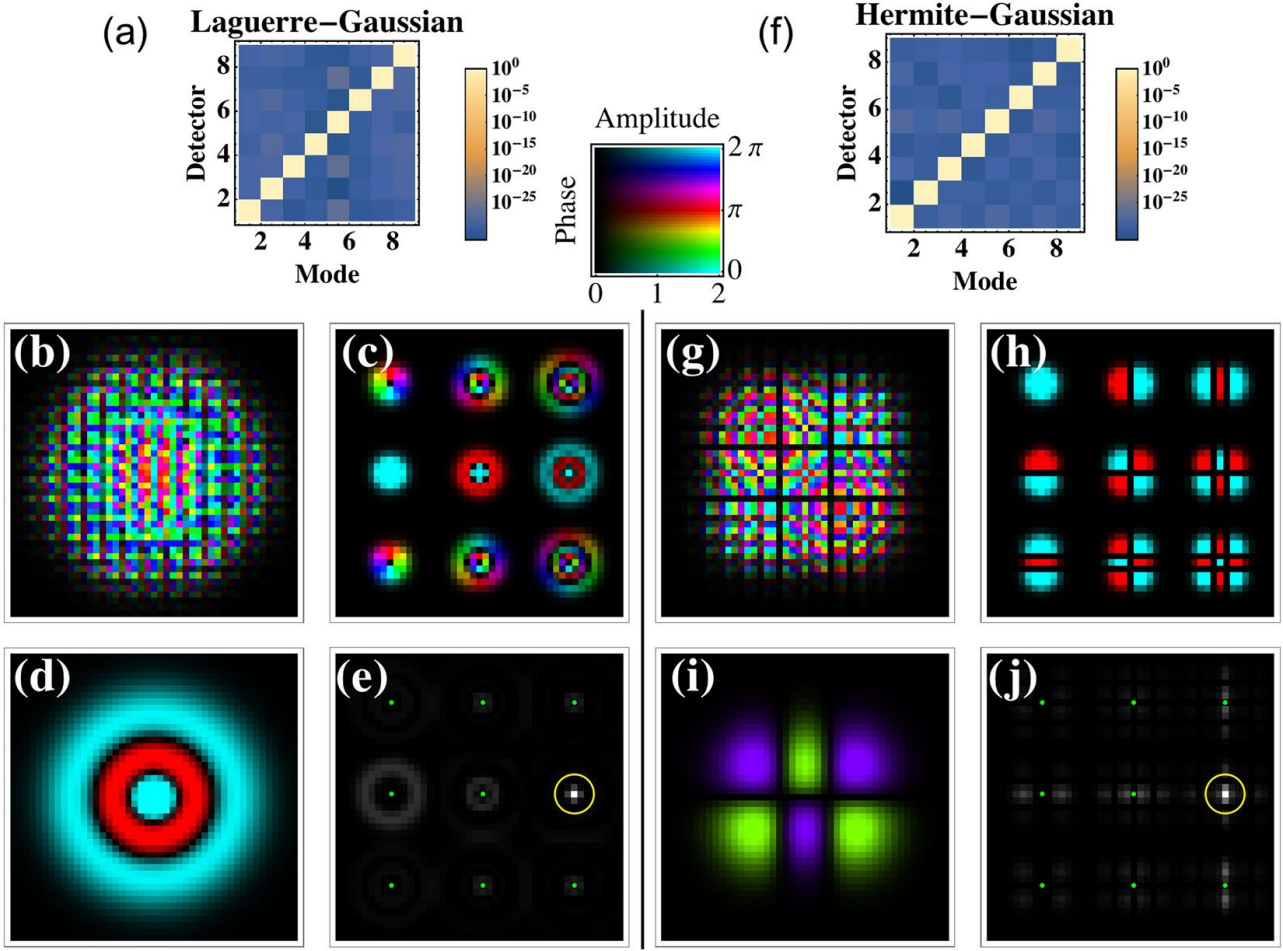
Decomposition of 9 Laguerre-Gaussian (**a**–**e**) and 9 Hermite-Gaussian (**f**–**j**) beams onto array of 9 single point detectors. (**a**,**f**) Detected intensity, in log scale, when array of detectors is illuminated by the corresponding modes. The intensity of each of the 9 detectors is displayed for each mode illumination. (**b**,**g**) Phase (hue) and amplitude (luminosity) of the mode splitting decomposition mask. Center inset shows the phase and amplitude color scheme used. (**c**,**h**) Fourier transform of the mode splitting decomposition mask. (**d**,**i**) Example incident mode and (**e**,**j**) corresponding output field. The center of the yellow circle is the position of the correct detector and the green dots represent the position of the other 8 detectors (Code available online)^27^.

Figure 1 shows the final masks, the detected signals from all detectors and an example output. We observe that the final mask delivers virtually no cross talk. However, from the example, it is apparent that the overall efficiency of the detection method is quite low. Indeed, in order to achieve low cross talk, much of the intensity of light is focused between the point like detectors. This efficiency is highly dependent on the position of the detectors, their sizes and the employed masks. While the mask does not show any clear features, its Fourier transform simplifies to a nine-fold convolution with mutually orthogonal, spatially separated, masks. The distance between these masks in the Fourier plane is directly related to the distance between the detectors used to determine the azimuthal and radial indices. The filter mask acts as a multi-output beamsplitter that after splitting the beam into 9 beamlets probes those with conjugated smaller masks each interfering constructively with only one of the incident modes. This is also visible in Fig. 1(e) where the Laguerre-Gaussian beam changes its azimuthal and radial order as a beamlet around each detector, while it transforms into a Gaussian beam only for the detector is specifically associated with the incident mode. The Fourier transform of the mode splitting eigenmask (Fig. 1(c,h)) show that this approach is, in this specific circumstance, similar to the carrier frequency modulation approach^23^ (CFM). To clarify the significance of the decomposition eigenmask and its relationship to the CFM approach, we study the eigenmask distinguishing between the different modes considered in Fig. 1 as we decrease the distance between the detectors in the array.

**Figure 2:**
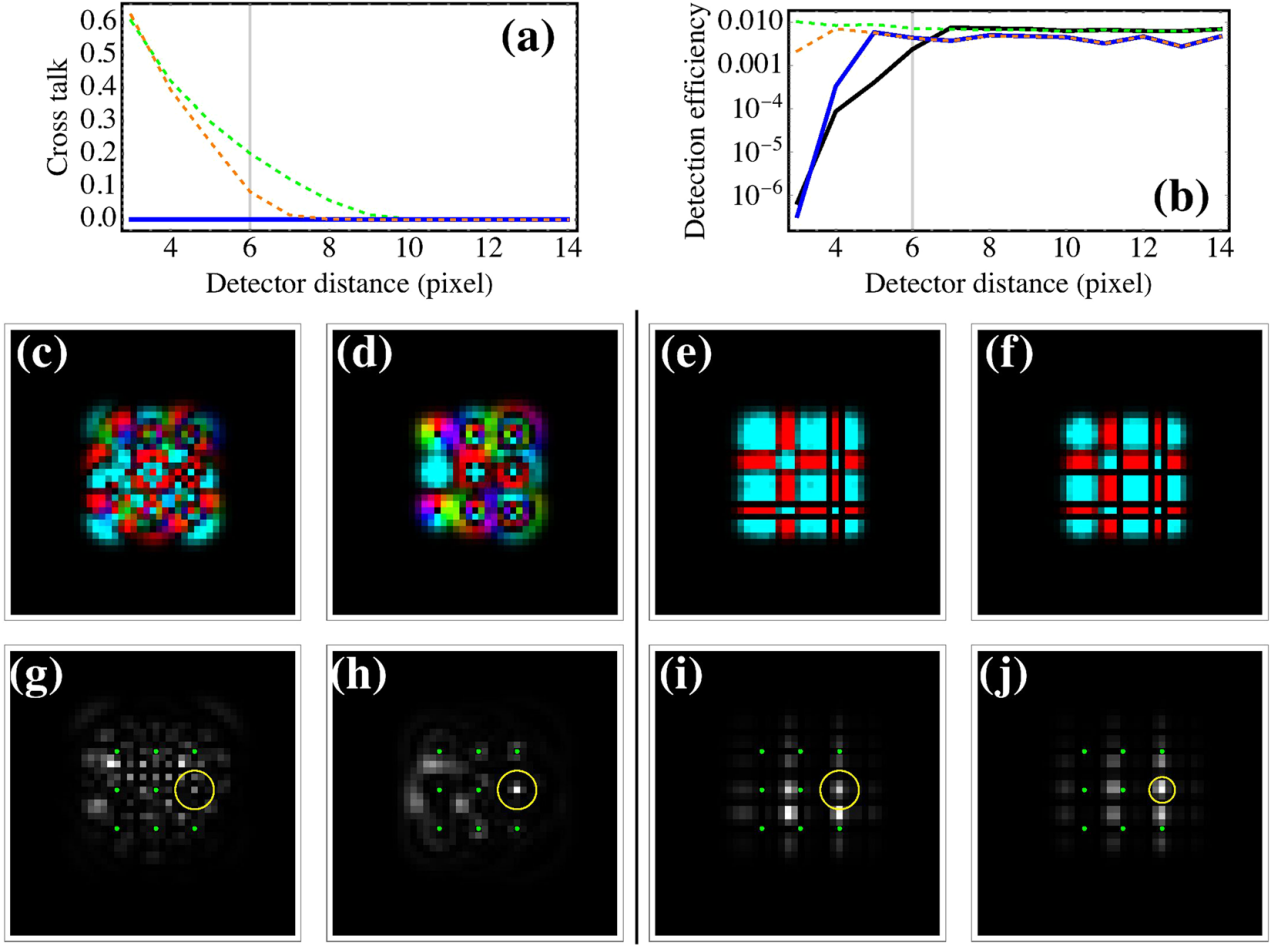
Comparison between the eigenmask method and carrier frequency modulation (CFM) approach^23^. (**a**) Cross talk as a function of detector distance for Laguerre-Gaussian (black curve: eigenmask; green dashed curve: CFM) and Hermite-Gaussian beams (blue curve: eigenmask; orange dashed curve: CFM). (**b**) Detection efficiency as a function of detector distance for Laguerre-Gaussian and Hermite-Gaussian beams (same coloring scheme as in part a). (**c**–**f**) Phase (hue) and amplitude (luminosity) of the Fourier transform of the decomposition mask and (**g**–**j**) example decomposition output intensity with (**c**,**g**) ${{\rm{LG}}}_{2}^{1}$ using the eigenmask (**d**,**h**) ${{\rm{LG}}}_{2}^{1}$ using CFM (**e**,**i**) HG_12_ using the eigenmask and (**f**,**j**) HG_12_ using CFM (Code available online)^27^.

Understanding the decomposition eigenmask as a superposition of smaller masks, each positioned in Fourier space at a detector’s center, offers a possible generalization of our result. Unfortunately, this generalization breaks down when the detector spacing is decreased and light rejected by one of the sub-masks reaches a different detector this will give rise to cross talk. It is only through the correct interference pattern that this cross talk can be eliminated and, as we will show, even used to increase the overall efficiency of the detection system. Figure 2 shows the case where the detector spacing is decreased to allow potential cross talk between nearest detectors. To quantify this behavior, we define the detection efficiency and cross talk as per Algorithm 1. The elimination of the cross talk is only possible through the use of the eigenmask decomposition which defines a set of masks that are mutually orthogonal with respect to the intensity interference on the 9 detectors. Without the eigenmask procedure, any increase in efficiency would be accompanied by an increase in cross talk delivering no overall improvement as is the case for the CFM method. Moreover, when using the eigenmask procedure, the cross talk is not affected by the spatial position of the detectors. Even randomly positioned detectors still offer a clear discrimination between the different transversal states at the expense of variable detection efficiency due to the variation in distance between the different detectors (see Supplementary Material).

## Discussion

In this paper, we demonstrated the decomposition of a beam into arbitrary orthogonal modes while minimizing cross talk. As an example, we studied numerically and experimentally the case of Laguerre-Gaussian and Hermite-Gaussian family of beams and their decomposition into detectable beams. So far our analysis has mainly considered classical field solutions; however, considering quantized fields may lead to important applications. For example, it may be desirable to measure the transverse mode content of a single photon source with respect to a chosen transverse mode basis set, e.g. that of the Hermite-Gaussian beams. In general, we are at liberty to choose any orthonormal basis set on the Hilbert space of the problem to analyze the quantum version of our method, but we argue that the optical eigenmodes are the most natural and economical basis. Recall that the optical system employed is composed of an initial field, classical or quantum, that impinges on a spatial filter mask, followed by free-space propagation and finally an array of point detectors that defines a region of interest on the output plane. This final detection stage restricts the field to the region of interest (ROI) on the output plane, in that light outside the ROI is lost, and this renders that optical system open in that not all intensity present in the input is available to trigger the detectors. In addition to being a orthonormal basis for the Hilbert space of problem the optical eigenmodes fully acknowledge the openness of the optical system due to the detectors, and are therefore candidates for a ‘pointer basis’ for the system. Indeed, a pointer basis represents a set of possible outcomes of measurements of a quantum system that correlate with a property of the quantum system (here the measurements at specific detectors of the array correlate with the transverse modes $\ell$). Zurek has discussed extensively how the pointer basis emerges naturally from the process of decoherence in which an open quantum system interacts with its environment^28^, here predominantly the detectors. So the optical eigenmodes present themselves a natural basis for the quantum theory of our method to determine the transverse mode content of quantized fields.

Turning now to the measurement of a single photon incident onto a single detector, the natural question arises as to its transversal mode. In particular, for quantized fields the signal measured by a photodetector can be described by 29–31:

13 $${\hat{m}}^{(I)}={\int }_{ROI}d\sigma \,{\hat{E}}^{(-)}({\bf{r}})\cdot {\hat{E}}^{(+)}({\bf{r}})$$

where ${\hat{E}}^{(-)}({\bf{r}})={\hat{a}}_{j}^{\dagger }{E}_{j}({\bf{r}}){e}^{i\omega t}$ and ${\hat{E}}^{(+)}({\bf{r}})={\hat{a}}_{k}{E}_{k}^{\ast }({\bf{r}}){e}^{-i\omega t}$ are the negative and positive frequency components parts of the electric field operator. Using the optical eigenmode basis as previously discussed this can be simplified to ${\hat{m}}^{(I)}={\sum }_{jk}{\hat{a}}_{k}^{\dagger }{M}_{jk}{\hat{a}}_{j}$ where ${M}_{jk}={\int }_{ROI}d\sigma {E}_{j}^{\ast }\cdot {E}_{k}$ with *E* representing the incident field. We can now define the quantum optical eigenmode field operators as ${\widehat{{\mathbb{E}}}}^{(-)}={\hat{{\mathbb{A}}}}_{j}^{\dagger }{{\mathbb{E}}}_{j}$ and ${\widehat{{\mathbb{E}}}}^{(+)}={\hat{{\mathbb{A}}}}_{k}{{\mathbb{E}}}_{k}^{\ast }$ with the optical eigenmode annihilation/creation operators given by:

14 $${\hat{{\mathbb{A}}}}^{\ell }=\frac{1}{\sqrt{{\lambda }^{\ell }}}\sum _{j}{({v}_{j}^{\ell })}^{\ast }{\hat{a}}^{j}\quad ;\quad {\hat{{\mathbb{A}}}}_{\ell }^{\dagger }=\frac{1}{\sqrt{{\lambda }_{\ell }}}\sum _{j}{v}_{\ell }^{j}{\hat{a}}_{j}^{\dagger }.$$

With these definitions the fundamental commutator $[{\hat{{\mathbb{A}}}}_{\ell }^{\dagger },{\hat{{\mathbb{A}}}}_{\ell }]=1$ is preserved. Finally, we deduce the detection operator as a function of the optical eigenmode creation/annihilation operators as

15 $${\hat{m}}^{(I)}=\sum _{j}{\hat{{\mathbb{A}}}}_{j}^{\dagger }{\hat{{\mathbb{A}}}}_{j}$$

which corresponds to the sum over the number operators in the optical eigenmode basis. For these eigenmode annihilation/creation operators to describe a single photon we need to be able to measure the eigenmode state of the light field for single photons and with single detectors. One way to achieve this is through the use of the transverse tomography using the eigenmasks described in the previous sections.

## Methods

### Experimental implementation

**Figure 3:**
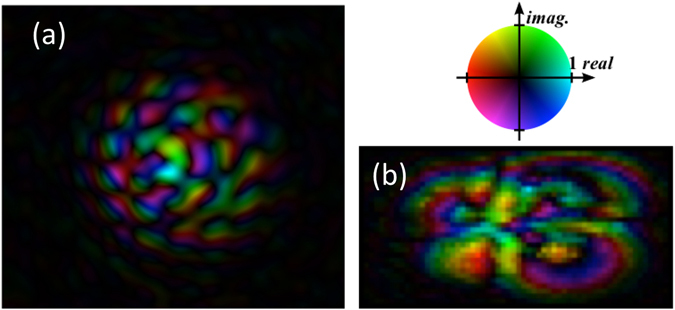
The experimental set-up consists of a Laguerre-Gaussian beam generating section (top), and a modal beam splitter section (bottom). The light field of the beam generator, shown in red, is assumed to be unknown when it connects to the input port of the modal beam splitter light path, shown in green to emphasize the distinction. L1-3: positive lenses of increasing focal length, PH: pinhole, LP: linear polarizer, *λ*/2: half-wave plate, ND: neutral density filter, DM: dielectric mirror, CCD: Charge Coupled Device, and A-SLM and P-SLM indicate amplitude and phase modulators respectively.

The proposed concept is demonstrated experimentally with the optical set-up shown in Fig. 3. The beam path can be separated into a section to generate a compound beam, and a section to create and test an eigenmask for the set of orthogonal input beams. The input beams are created using a standard amplitude and phase modulation setup using a dual head reflective spatial light modulator (SLM, Holoeye HEO 1080 P), and imaged using a magnifying telescope onto the SLM of the eigenmask in the second section of the beam path. The active area of the beam creation SLM (A-SLM1 and P-SLM1 combined) and the focal lengths are chosen such that the beam illuminates a significant area of the eigenmask SLM (A-SLM2 and P-SLM2 combined).

Before the eigenmask can be calculated for SLM2, the optical system must be characterized for each of the input beams. To this end an image stack is acquired while a sequence of 1800 probe masks is displayed on SLM2 for each of the input beams. Each of the 1800 beams are interfered with a reference beam, enabling the measure of the relative phase and amplitude of the beam created be each probe mask^32^. The probe masks are chosen so that the image plane field at the CCD can be readily calculated for any linear combination of probe masks and for any input beam. Consequently, the eigenmask is calculated as defined above.

**Figure 4:**
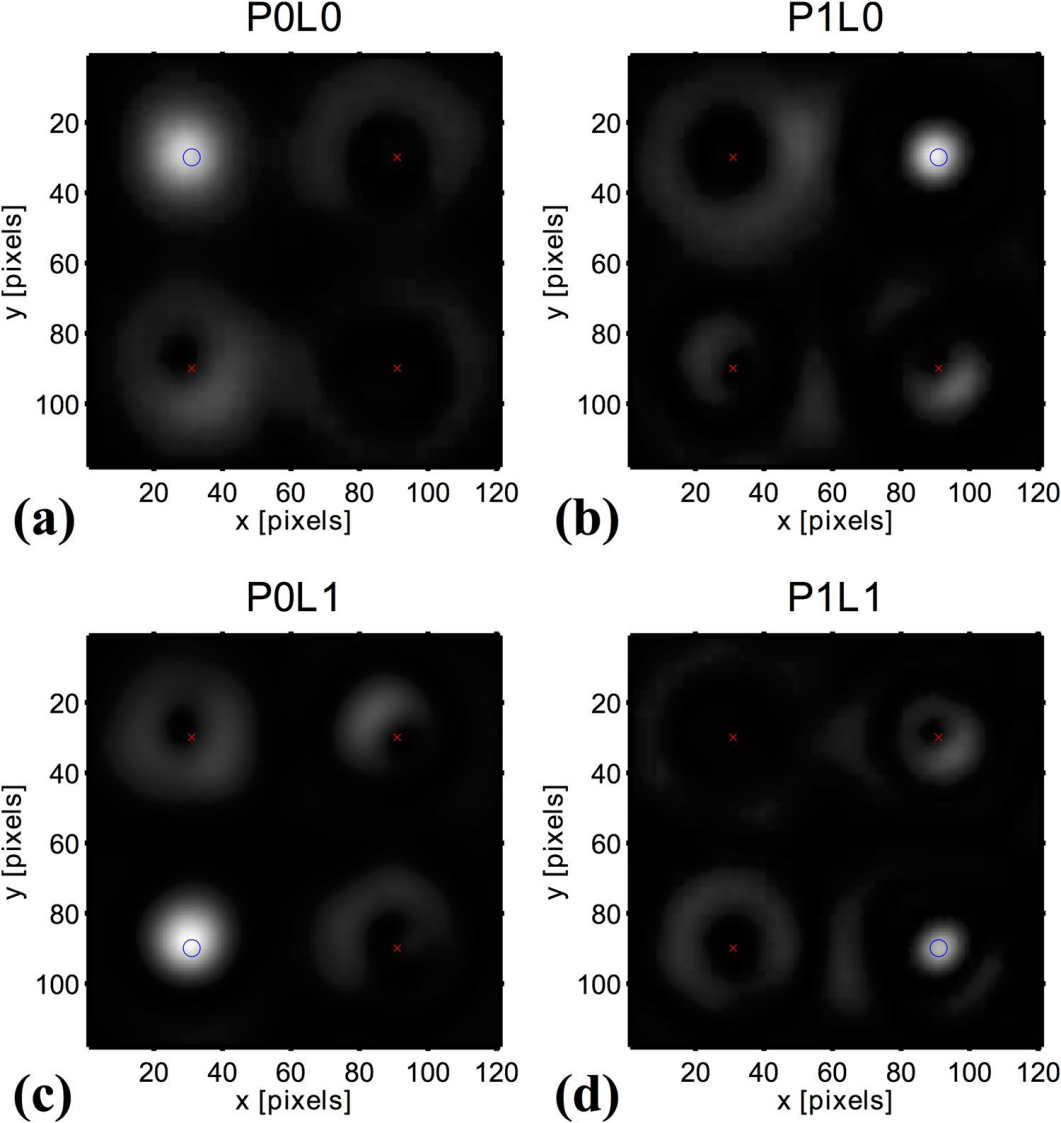
The eigenmask calculated from the measurements, (**a**) in the spatial domain as sent to the spatial light modulator, and (**b**) its Fourier transform. The intensity and hue indicate respectively the amplitude and argument of the complex values.

**Figure 5:**
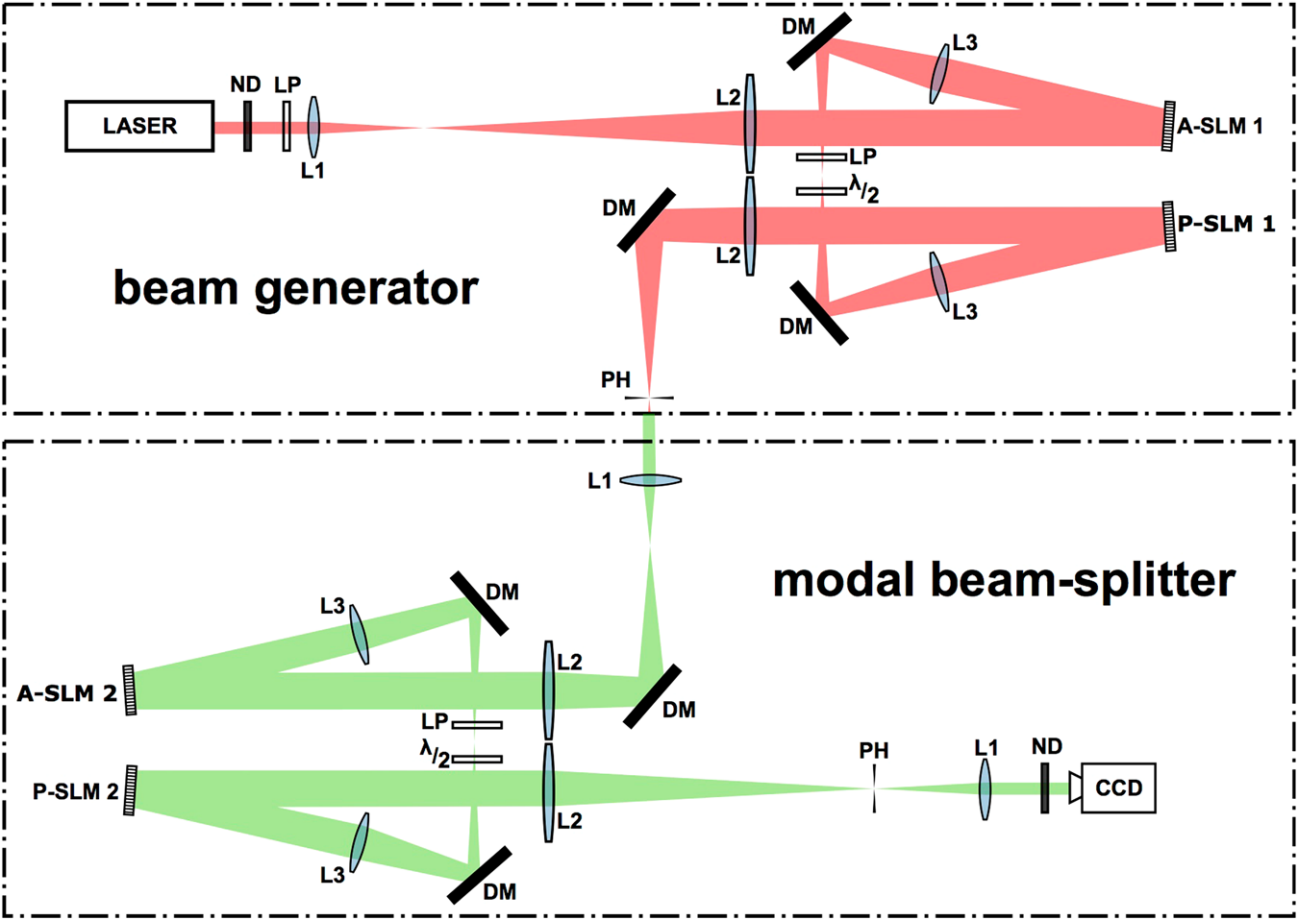
Experimental measurements of four Laguerre-Gaussian beams filtered with the single eigenmask. Sub plots (**a**–**d**) show the recorded image for the respective beams (P-number, L-number): (0, 0), (1, 0), (0, 1), and (1, 1). Red crosses mark the positions that should not be irradiated, blue circles indicate the positions that of unity irradiance.

The procedure is demonstrated with four orthogonal Laguerre Gaussian beams having *p*-numbers 0 and 1, and $\ell$-numbers 0 and 1. Four points on the active area of the CCD are chosen as the target detectors. Figure 4 shows the obtained eigenmask respectively in the spatial and in the spatial frequency domain. It can be noticed from Fig. 4(a) that the mask has only significant values in a central circular area around the beam waist of the four input beams. Less structure can be seen in the phase of the eigenmask. Figure 4(b) shows the Fourier transform of the eigenmask, only 1800 (60 × 30) non-zero values are present since the Fourier components were chosen as probes. Four different regions, corresponding to the four detector points can be discerned in the Fourier transform of Fig. 4(b). Figure 5 shows the experimental images obtained when loading the eigenmask on SLM2, and recording the image plane intensity for the four input beams. The experimental results can be seen to fit well with the semi-theoretical calculations with modest drift due to mechanical instabilities in the optical path. As a result, some crosstalk should be expected between the detectors (see Supplementary Material).

## Electronic supplementary material

Supplementary material
