# Supplementary material for: Modal beam splitter: determination of the transversal components of an electromagnetic light field
Source: Sci Rep. 2017 Aug 22;7:9139. doi: 10.1038/s41598-017-08657-9 (PMC5567242; doi:10.1038/s41598-017-08657-9)
Supplement: Supplementary file 1 — Supplementary material [file 41598_2017_8657_MOESM1_ESM.pdf]

Supplementary material: Modal beam splitter: determination of the transversal components of an electromagnetic light field

Michael Mazilu*[1], Tom Vettenburg[1], Martin Ploschner[1], Ewan M. Wright[2] and Kishan Dholakia[1,2]

[1]SUPA, School of Physics and Astronomy, University of St Andrews, St Andrews, Fife, KY16 9SS, UK
[2]College of Optical Sciences, The University of Arizona, 1630 East University Boulevard, Tucson, AZ 85721-0094, USA

July 13, 2017

1 Detection efficiency

To improve the detection efficiency, one can consider a system of cascaded mask and detector pairs each working to detect a single mode. One possibility is to embed the detector in the middle of a mirror such that it perfectly detects a single mode while all other modes are reflected towards the next filter mask in the chain. Figure 1 shows the effect of the single layer decomposition mask which focuses a single mode onto a single detector. This approach delivers the maximum theoretical detection efficiency provided that the filter mask is lossless, hence phase-only. Indeed, a phase only mask will lead to all spatial components of the mode in question to constructively interfere in a point and due to energy conservation this automatically means that all other modes are excluded from this focal point. Overall, a cascaded multistage detection is equivalent to a unitary transformation where each stage corresponds to a one-dimensional projection. Figures 1a (random detector positions) and 1b (regular detector positions) also illustrate the positioning effect of the detectors.

*michael.mazilu@st-andrews.ac.uk

2 Semi-theoretical simulation

If the system is measured with an appropriate number of probe masks, it is possible to predict, based on the experimental measures, the performance of the eigenfilter for the input beams. Figure 2 shows the simulated images for the four input beams. These images take into account the experientially measured probe masks. The positions of the detectors are marked with a blue circle if the eigenmask is designed to illuminate the specific detector, and with red crosses if the eigenmask is designed to not illuminate the specific detectors. For the four beams, low irradiance can be seen in the regions around three detectors, and high irradiance can be seen near the fourth detector. The irradiance is not always maximized at the target detector; however, unit irradiance is obtained at the target detectors for the four input beams.

3 Crosstalk

Figure 3 shows the experimental crosstalk matrix for the four input beams and the four detectors. In absence of imperfections, the off-diagonal element should be identical to zero, and the on-diagonal elements should be identical to one another.

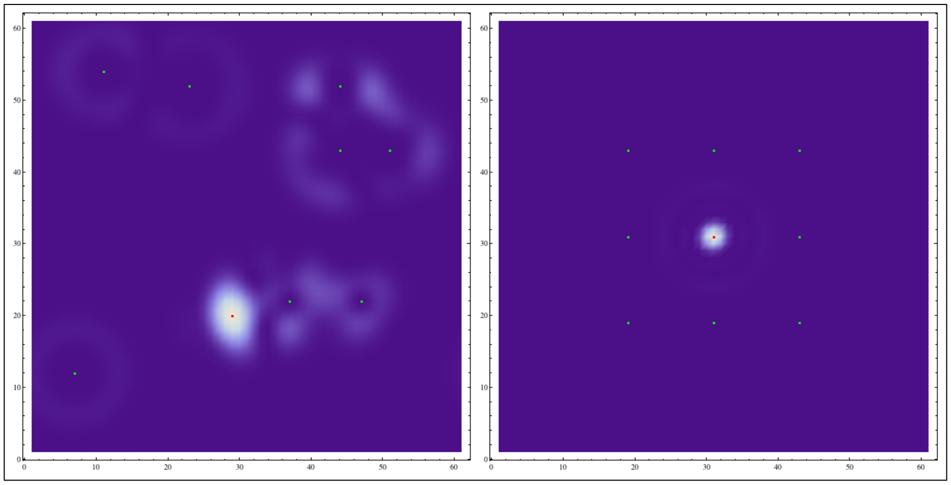

Figure 1: (a) Output field with dots showing the position of the nine randomly positioned detectors. The incident beam is LG_0^0 and its associated detector is highlighted in red and in green are all the other detectors. (b) LG_1^1 incident beam for the single detector case and regular detector grid.

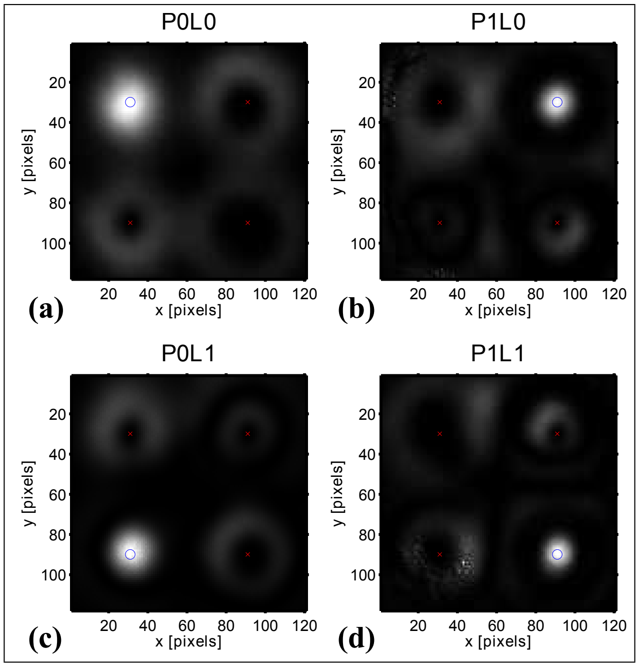

Figure 2: Semi-theoretical image of four Laguerre-Gaussian beams filtered with the single eigenmask is simulated for experimentally obtained probes. Subplots (a)-(d) show the recorded image for the respective beams (p-number,ℓ-number): (0,0), (1,0), (0,1), and (1,1). Red crosses mark the positions that should not be irradiated, blue circles indicate the positions that of unity irradiance.

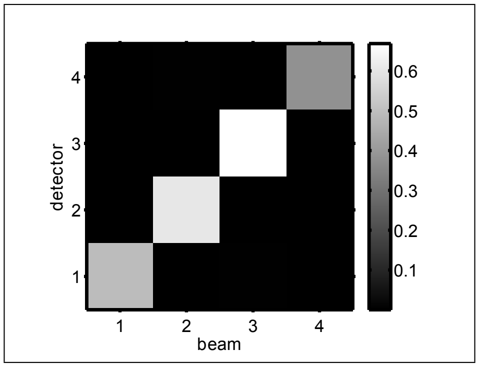

Figure 3: Experimental crosstalk of the four input beams at the four detectors. The beams and detectors are ordered as follows: (p-number,ℓ-number)=(0,0), (1,0), (0,1), and (1,1).
